# Renal Function and 4‐Year Outcomes in Edoxaban‐Treated Patients: A Sub‐Analysis From the ETNA‐AF Europe Study

**DOI:** 10.1111/eci.70268

**Published:** 2026-10-28

**Authors:** Raffaele De Caterina, Eva‐Maria Fronk, Joana Nunes, Mathias Lamparter, Paulus Kirchhof, Carmine Zoccali, the ETNA‐AF Europe Investigators

**Affiliations:** ^1^ University of Pisa and Pisa University Hospital Pisa Italy; ^2^ Daiichi Sankyo Europe GmbH Munich Germany; ^3^ Institute of Cardiovascular Sciences University of Birmingham Birmingham UK; ^4^ Institute of Molecular Biology and Genetics Ariano Irpino Italy; ^5^ Association for Hypertension, Nephrology, and Kidney Transplant (IPNET), Nephrology Great Metropolitan Hospital Reggio Calabria Italy

**Keywords:** atrial fibrillation, chronic kidney disease, edoxaban, non‐vitamin K oral anticoagulant, stroke prevention

## Abstract

**Background:**

Non‐vitamin K oral anticoagulants, like edoxaban, are essential in managing atrial fibrillation (AF). The impact of renal function on edoxaban dosages in real‐world settings remains unclear. This 4‐year ETNA‐AF‐Europe study follow‐up analysis investigated how chronic kidney disease (CKD) stages affect the safety and effectiveness of usual edoxaban doses.

**Methods:**

ETNA‐AF‐Europe (NCT02944019) was an observational study conducted from 2016 to 2022 across 776 centres in 10 European countries. Hazard ratios (HR) and annualized event rates were calculated for all‐cause death, cardiovascular (CV) death, stroke and bleeding events for the overall population and participants stratified by dose (30/60 mg) and CKD stages at baseline.

**Results:**

Of the 13,164 participants in the full analysis set, 9114 participants with CKD (Stage 1, *n* = 2064; Stage 2, *n* = 4855; Stage 3, *n* = 1985; Stage 4, *n* = 135; missing, *n* = 75) received label‐recommended edoxaban doses. Compared with CKD Stage 1, higher CKD stages were associated with increased risks for stroke (Stage 3: HR [95% CI]: 1.889 [1.240–2.878], *p* = 0.003) and CV death (HR [95% CI]; Stage 4: 14.450 [8.270–25.247], Stage 3: 4.672 [3.312–6.969], Stage 2: 2.066 [1.394–3.062], all *p* < 0.001). All‐cause death followed a similar trend. After adjusting for age, sex, body weight, CHA_2_DS_2_‐VASc and modified HAS‐BLED scores, most bleeding and effectiveness outcomes showed no nominally significant differences across CKD stages.

**Conclusions:**

Edoxaban in patients with AF is effective and well tolerated across CKD stages and appropriate label dosing can offset the risk from renal impairment.

## Graphical abstract

The ETNA‐AF‐Europe 4‐year follow‐up sub‐analysis reaffirms the effectiveness and safety of adherent label‐recommended doses of edoxaban in patients with AF with varying degrees of renal impairment. Low number of patients and events of ^¶^stroke (*n* = 2) and ^†^ICH (*n* = 1) may warrant results to be interpreted with caution. *Adjusted hazard ratio statistically significant. AF, atrial fibrillation; CKD, chronic kidney disease; CV, cardiovascular; ICH, intracranial haemorrhage.

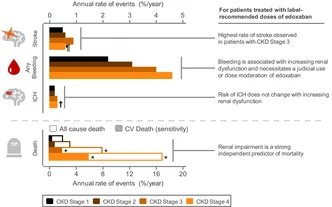

## 1 Introduction

Atrial fibrillation (AF) is a prevalent cardiac arrhythmia associated with increased risks of stroke and systemic embolism [1]. Effective oral anticoagulation (OAC) is crucial to reducing these risks, with non‐vitamin K oral anticoagulants (NOACs), such as edoxaban, playing a pivotal role in AF management [2, 3, 4, 5]. However, patient characteristics can influence the safety and effectiveness of NOACs. Notably, varying renal function among AF patients, due to the high prevalence of chronic kidney disease (CKD) in this usually elderly population, increases the risk of thromboembolic events or bleeding [2, 6]. Consequently, understanding how renal function impacts the therapeutic profile of summary of product characteristics (SmPC: label) recommended edoxaban dosages in real‐world settings is critical to optimizing anticoagulation strategies [7].

The ETNA‐AF‐Europe (NCT02944019), a prospective observational study, has provided extensive real‐world evidence on the use of edoxaban in a diverse European AF population [8, 9, 10]. The 4‐year follow‐up highlighted the long‐term tolerability and effectiveness of edoxaban in patients with AF, showing relatively low annualized rates of death, stroke, major bleeding and intracranial haemorrhage (ICH) [11]. The collected data offered a valuable opportunity to investigate the influence of CKD stages on the safety and effectiveness of edoxaban dosages. After 2 years of follow‐up, patients with AF who also met the criteria of ‘worsening renal function’ showed nominally significantly higher annualized rates of all‐cause death, cardiovascular (CV) death and major bleeding. The strongest reported predictors of worsening renal function were a high CHA_2_DS_2_‐VASc score, high baseline creatinine clearance, low body weight and older age [12].

In this 4‐year follow‐up sub‐analysis, in addition to reporting patient characteristics and clinical outcomes within CKD subgroups at baseline, we examine the baseline data and annualized event rates of key efficacy and safety parameters from ETNA‐AF‐Europe to comprehend real‐world findings. Accordingly, we aim to bridge the gap between clinical trial data and real‐world evidence, offering insights into how recommended edoxaban dosages perform across categories of renal function impairment.

## 2 Methods

### 2.1 Study Design and Participants

ETNA‐AF‐Europe (NCT02944019) was a prospective, multinational, multi‐centre, post‐authorization, observational study that ran from 2016 to 2022 and was conducted across 776 centres in 10 European countries (Austria, Belgium, Germany, Ireland, Italy, the Netherlands, Portugal, Spain, Switzerland and the United Kingdom) [9, 11]. The study was approved by the Institutional Review Boards and Independent Ethics Committees for participating centres in compliance with Guidelines for Good Pharmacoepidemiologic Practice (GPP). Detailed study design has been published previously [9]. The ETNA‐AF Europe study had broad inclusion and exclusion criteria. Patients were included if they provided written informed consent, were treated with edoxaban for AF according to edoxaban SmPC and were not simultaneously participating in any other interventional study. The decision to treat patients with edoxaban was made entirely by the physician. Nevertheless, when selecting patients for inclusion, investigators were expected to comply with SmPC dosing recommendations based on renal function. No explicit exclusion criteria were provided to avoid selection bias and to allow for documentation of routine clinical practice.

Patients with AF treated with edoxaban at baseline were followed up every 12 ± 2 months for 4 years. Patients who permanently discontinued edoxaban were followed annually for a further 2 years after discontinuation or until the end of the observational period, whichever came first.

### 2.2 Objectives and Outcome Measures

In this post hoc analysis of the final 4‐year follow‐up data (ETNA‐AF‐Europe 4‐year follow‐up), the primary objective was to evaluate real‐world effectiveness and safety data in participants who were on the label‐recommended edoxaban doses (30/60 mg) stratified according to their renal function, calculated utilizing the CKD staging (estimated glomerular filtration rate [eGFR]) according to the CKD‐EPI formula; Stage 1: eGFR ≥ 90 mL/min/1.73 m^2^, Stage 2: eGFR ≥ 60 to < 90 mL/min/1.73 m^2^, Stage 3: eGFR ≥ 30 to < 60 mL/min/1.73 m^2^, Stage 4: eGFR ≥ 15 to < 30 mL/min/1.73 m^2^ [7, 13].

Effectiveness outcomes included stroke, transient ischemic attack (TIA) and systemic embolism event (SEE). Safety outcomes included all‐cause death and CV death (sensitivity [death due to CV cause or bleeding, or if a stroke, TIA, SEE, PE, MI, VTE or major bleeding occurred within 30 days before death and the reason of death is unknown or missing]), bleeding events, including gastrointestinal (GI) bleeding, and ICH. Major bleeding, clinically relevant non‐major bleeding, strokes, SEE and deaths were adjudicated by a clinical event adjudication committee (bleeding events were adjudicated using the International Society on Thrombosis and Haemostasis definitions). Of note, definitions of both any stroke and ICH included haemorrhagic stroke events; thus, they were counted as part of both these outcomes.

Besides reporting descriptions of patients' characteristics and outcomes from the aforementioned groups, baseline data and annualized event rates from ETNA‐AF‐Europe were descriptively set into perspective with the annualized event rates reported for patients included in the randomized ENGAGE TIMI trial [11, 14].

### 2.3 Statistical Analysis

Baseline characteristics were summarized descriptively as frequencies (*n*, %) or mean ± standard deviation (SD), whilst safety and effectiveness outcomes were presented as annualized event rates for the overall population and the subgroups. Annualized event rates for each endpoint (%/year, equivalent to the number of first events per 100 patient years) were calculated as annual rate = 100 × number of patients with at least one event within 4 years after baseline ÷ sum of all individual patient observation time from reference start date until first event or if patient completed/discontinued from the study without experiencing an event within 4 years after baseline, until date of premature study discontinuation, or death, or last available follow‐up date, or censoring within 48 months from baseline data collection point, whichever occurred first [years]. Percentage calculations excluded patients with missing or unknown data. Patients with missing eGFR and patients on off‐label dose or off‐label CKD stage were excluded from the analysis (Figure 1), and missing or incomplete dates were imputed. Frailty data in ETNA‐AF Europe was an optional binary capture [15], categorized as yes/no/unknown, as perceived by physicians as a personal judgement, without any mandated protocol.

**FIGURE 1:**
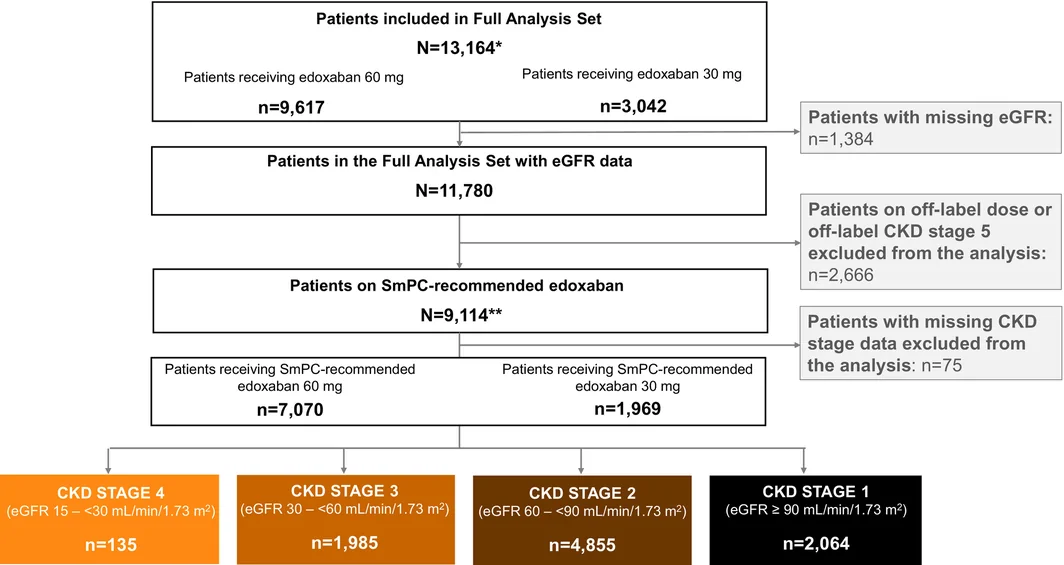
Patient flow diagram (full analysis set of ETNA‐AF Europe and full analysis set of patients considered in this sub‐analysis). *Missing *n* = 505 patients with missing dose data. **Seventy‐five patients with missing CKD stage data. Definition of CKD stages (eGFR, CKD‐EPI Formula): Stage 4: eGFR ≥ 15 to < 30 mL/min/1.73 m^2^, Stage 3: eGFR ≥ 30 to < 60 mL/min/1.73 m^2^, Stage 2: eGFR ≥ 60 to < 90 mL/min/1.73 m^2^, Stage 1: eGFR > 90 mL/min/1.73 m^2^. CKD, chronic kidney disease; eGFR, estimated glomerular filtration rate; SmPC, summary of product characteristics.

Outcomes data were reported for the full analysis set. Annualized event rates were calculated for all‐cause death, CV death (sensitivity), any stroke, any bleeding, major bleeding, ICH, any GI bleeding and major GI bleeding in the overall population in line with the label recommendations, and stratified by 30 and 60 mg dose at baseline [7].

Hazard ratios (HRs) and *p*‐values for effectiveness and safety outcomes were calculated based on Cox‐regression without any adjustment and referred to the comparison of the different renal stages versus the reference CKD Stage 1 (≥ 90 mL/min). HR and 95% CI are not displayed for groups with < 5 observations. HRs were further adjusted for confounding factors at baseline, including age, sex, body weight and clinical risk scores (CHA_2_DS_2_‐VASc and modified HAS‐BLED; Section S1).

Both non‐adjusted and adjusted HRs are presented in the results and highlight the effect of confounding variables. No adjustments were made for multiple testing; therefore, all results are descriptive and should be interpreted accordingly. The statistical analysis was performed using SAS version 9.4 (SAS Institute, Cary, NC, USA).

## 3 Results

### 3.1 Baseline Characteristics per CKD Stage

ETNA‐AF‐Europe enrolled 13,632 participants, 13,164 of whom were part of the full analysis set, with 11,780 patients having eGFR data, showing worsening renal function across CKD stages (Figure 1, Section S2 and Table S1). Among them, 9114 participants were on label‐recommended edoxaban doses with 4‐year follow‐up, divided across classes of worsening renal function: Stage 1 (*n* = 2064), Stage 2 (*n* = 4855), Stage 3 (*n* = 1985) and Stage 4 (*n* = 135). Seventy‐five enrolled patients had missing CKD stage (Figure 1). Participants were predominantly male (57.8%), with mean age of 73.3 years, and 48.8% were aged ≥ 75 years. Baseline renal function was associated with older age, lower body weight, more frequent dose reductions, increased perceived frailty and a higher prevalence of CV risk factors (Table 1). Dose‐reduction criteria for edoxaban, based on creatinine clearance (CrCl) or body weight ≤ 60 kg, were common in patients with CKD Stages 3 and 4 (CrCl: 63.6% and 100.0%, respectively; body weight ≤ 60 kg: 16.5% and 14.4%, respectively). CV risks also differed across CKD stages. CHA_2_DS_2_‐VASc and modified HAS‐BLED scores, indicative of stroke and bleeding risks, were highest in CKD Stage 4 and progressively decreased with better renal function (Table 1). Hypertension was the most prevalent CV comorbidity (76.8% overall), especially in CKD Stage 4 (88.1%). CKD 4 stage patients had the greatest % of patients with previous history of diabetes mellitus (46.7%). Prior CV and bleeding events, including ischemic stroke and major bleeding, were more common in advanced CKD stages.

**TABLE 1:** Baseline characteristics by renal CKD stages for all participants from ETNA‐AF Europe 4‐year follow‐up that were on label‐recommended edoxaban dose (30 or 60 mg) (full analysis set, *N* = 9114).^a^

	Total	CKD Stage 4	CKD Stage 3	CKD Stage 2	CKD Stage 1
*N* (%)	9114 (100.0)	135 (1.5)	1985 (22.0)	4855 (53.7)	2064 (22.8)
Male, *n* (%)	5271 (57.8)	44 (32.6)	938 (47.3)	2886 (59.4)	1386 (67.2)
Age (years), mean (SD)	73.3 (9.6)	79.9 (7.9)	79.1 (7.7)	73.6 (8.6)	66.4 (9.3)
≥ 75 years old, *n* (%)	4448 (48.8)	107 (79.3)	1482 (74.7)	2436 (50.2)	370 (17.9)
Body weight (kg), mean (SD)	81.5 (17.1)	76.8 (15.5)	77.9 (16.4)	81.7 (16.5)	85.4 (18.3)
BMI (kg/m^2^), mean (SD)	28.2 (5.0)	28.0 (4.7)	27.9 (5.0)	28.1 (4.9)	28.8 (5.4)
Patients with dose reduction criteria, *n* (%)					
CrCl < 50 mL/min	1698 (18.7)	135 (100.0)	1262 (63.6)	293 (6.0)	1 (< 0.1)
Body weight ≤ 60 kg	810 (8.9)	22 (16.5)	283 (14.4)	373 (7.7)	86 (4.2)
Concomitant use of P‐gp inhibitors	114 (1.3)	5 (3.7)	59 (3.0)	35 (0.7)	4 (0.2)
Edoxaban 60 mg, *n* (%)	7082 (77.7)	0	708 (35.7)	4387 (90.4)	1975 (95.7)
CHA_2_DS_2_‐VASc (cal.), mean (SD)	3.2 (1.5)	4.5 (1.2)	4.0 (1.4)	3.1 (1.4)	2.4 (1.3)
CHA_2_DS_2_‐VASc = 0, *n* (%)	191 (2.1)	0	5 (0.3)	70 (1.5)	116 (5.7)
CHA_2_DS_2_‐VASc = 1, *n* (%)	921 (10.3)	0	51 (2.6)	459 (9.6)	409 (20.3)
CHA_2_DS_2_‐VASc ≥ 2, *n* (%)	7796 (87.5)	131 (100.0)	1873 (97.1)	4228 (88.9)	1493 (74.0)
HAS‐BLED (cal.), mean (SD)	2.5 (1.0)	3.2 (0.7)	3.1 (0.7)	2.5 (1.0)	1.7 (0.9)
Physician‐categorized frailty, *n* (%)	1028 (11.3)	44 (32.6)	450 (22.7)	443 (9.1)	86 (4.2)
History of CV disease					
Hypertension	7002 (76.8)	119 (88.1)	1659 (83.6)	3660 (75.4)	1502 (72.8)
Congestive heart failure	1495 (16.4)	47 (34.8)	506 (25.5)	698 (14.4)	234 (11.3)
Myocardial infarction	401 (4.4)	8 (5.9)	122 (6.1)	195 (4.0)	74 (3.6)
Valvular disease	1332 (14.6)	40 (29.6)	409 (20.6)	664 (13.7)	209 (10.1)
Peripheral artery disease	285 (3.1)	9 (6.7)	107 (5.4)	123 (2.5)	44 (2.1)
History of diabetes mellitus, *n* (%)	2035 (22.3)	63 (46.7)	577 (29.1)	959 (19.8)	414 (20.1)
History of stroke and ICH, *n* (%)					
Ischemic stroke	481 (5.3)	10 (7.4)	122 (6.1)	258 (5.3)	89 (4.3)
Stroke, unknown	51 (0.6)	1 (0.7)	15 (0.8)	24 (0.5)	10 (0.5)
Transient ischemic attack	314 (3.4)	2 (1.5)	81 (4.1)	173 (3.6)	56 (2.7)
Intracranial haemorrhage	44 (0.5)	1 (0.7)	17 (0.9)	19 (0.4)	7 (0.3)
Previous history of bleeding					
Any bleeding	278 (3.1)	6 (4.4)	98 (4.9)	131 (2.7)	39 (1.9)
GI bleeding (major or CRNM)	69 (0.8)	3 (2.2)	34 (1.7)	27 (0.6)	4 (0.2)
Major	78 (0.9)	2 (1.5)	30 (1.5)	34 (0.7)	11 (0.5)
CRNM	91 (1.0)	2 (1.5)	39 (2.0)	41 (0.8)	8 (0.4)
Minor	111 (1.2)	2 (1.5)	29 (1.5)	57 (1.2)	21 (1.0)
Current AF type					
Paroxysmal	4780 (52.5)	64 (47.4)	915 (46.1)	2583 (53.3)	1169 (56.7)
Persistent	2311 (25.4)	31 (23.0)	476 (24.0)	1254 (25.9)	539 (26.2)
Long/standing persistent	224 (2.5)	7 (5.2)	57 (2.9)	107 (2.2)	53 (2.6)
Permanent	1787 (19.6)	33 (24.4)	535 (27.0)	904 (18.6)	300 (14.6)

*Note:* Definition of CKD stages (eGFR, CKD‐EPI Formula): Stage 4: eGFR ≥ 15 to < 30 mL/min/1.73 m^2^, Stage 3: eGFR ≥ 30 to < 60 mL/min/1.73 m^2^, Stage 2: eGFR ≥ 60 to < 90 mL/min/1.73 m^2^, Stage 1: eGFR ≥ 90 mL/min/1.73 m^2^. CHA2DS2‐VASc, congestive heart failure (1 point), hypertension (1 point), age ≥ 75 (2 points), diabetes (1 point), stroke/TIA/SEE (2 points), vascular disease (1 point), age 65–74 (1 point) and female sex (1 point); HAS‐BLED, hypertension (1 point), CrCl ≤ 80 mL/min or liver disease (1 or 2 points), stroke history (1 point), bleeding history or predisposition (1 point), age of > 65 years (1 point), medication usage predisposing to bleeding or alcohol usage (1 or 2 points); P‐gp, P‐glycoprotein 1 inhibitors requiring edoxaban dose reduction (cyclosporine, dronedarone, erythromycin, ketoconazole).

Abbreviations: AF, atrial fibrillation; BMI, body mass index; cal., calculated; CKD, chronic kidney disease; CrCl, derived creatinine clearance (Cockgroft‐Gault); CRNM, clinically relevant non‐major; CV, cardiovascular; eGFR, estimated glomerular filtration rate (CKD‐EPI); GI, gastrointestinal; ICH, intracranial haemorrhage; SD, standard deviation; SEE, systemic embolism event; TIA, transient ischemic attack.

^a^ Category of missing CKD is not shown.

### 3.2 Effectiveness Outcomes per CKD Stages

The annualized event rates for patients in ETNA‐AF‐Europe who were on label‐recommended edoxaban doses varied across CKD stages (Table 2). Stroke rates were highest in CKD Stage 3 (0.9% per year [95% CI: 0.70–1.18]). SEEs and TIAs followed an increasing trend, with the lowest rates in CKD Stage 1 and highest rates in CKD Stage 4 (0.6% and 0.9% per year [95% CI: 0.14–2.27 and 0.28–2.66], respectively). When compared to patients with CKD Stage 1, worsening renal function was associated with statistically significant increased risks (at a nominal level) for stroke for Stage 3 (HR [95% CI]: 1.889 [1.240–2.878], *p* = 0.003; Figure 2, left panel). No statistically robust difference in risk versus Stage 1 patients was observed for any of the outcomes when data were adjusted for age, sex, body weight and clinical risk scores (adjusted HR: Figure 2, right panel). No valid conclusion can be drawn for TIA and SEE for Stage 4, as the number of observations (3 observations for TIA and 2 observations for SEE) was too small within that group.

**TABLE 2:** Absolute and relative frequencies, annual rates and confidence intervals of outcomes of interest by renal stages in all patients from ETNA‐AF Europe 4‐year follow‐up that were on label‐recommended edoxaban dose (30 mg [*N* = 2032] or 60 mg [7082]) (full analysis set, *N* = 9114).^a^

	CKD Stage 4 (*N* = 135)		CKD Stage 3 (*N* = 1985)		CKD Stage 2 (*N* = 4855)		CKD Stage 1 (*N* = 2064)	
	*n* (%)	Annual rates, % [95% CI]	*n* (%)	Annual rates, % [95% CI]	*n* (%)	Annual rates, % [95% CI]	*n* (%)	Annual rates, % [95% CI]
Stroke	2 (1.5)	0.6 [0.14, 2.28]	57 (2.9)	0.9 [0.70, 1.18]	97 (2.0)	0.6 [0.47, 0.70]	35 (1.7)	0.5 [0.34, 0.67]
TIA	3 (2.2)	0.9 [0.28, 2.66]	25 (1.3)	0.4 [0.27, 0.59]	52 (1.1)	0.3 [0.23, 0.40]	16 (0.8)	0.2 [0.13, 0.36]
SEE	2 (1.5)	0.6 [0.14, 2.27]	7 (0.4)	0.1 [0.05, 0.23]	5 (0.1)	0.0 [0.01, 0.07]	3 (0.1)	0.0 [0.01, 0.13]
CV death (sensitivity)	21 (15.6)	5.9 [3.86, 9.08]	121 (6.1)	1.9 [1.59, 2.28]	143 (2.9)	0.8 [0.71, 0.99]	30 (1.5)	0.4 [0.28, 0.58]
All‐cause death	60 (44.4)	16.9 [13.14, 21.79]	503 (25.3)	7.9 [7.26, 8.64]	526 (10.8)	3.1 [2.84, 3.37]	148 (7.2)	2.0 [1.71, 2.36]
Any bleeding	15 (11.1)	4.6 [2.75, 7.58]	240 (12.1)	4.0 [3.56, 4.59]	494 (10.2)	3.1 [2.83, 3.37]	156 (7.6)	2.2 [1.89, 2.59]
Major bleeding	7 (5.2)	2.0 [0.97, 4.25]	92 (4.6)	1.5 [1.20, 1.81]	141 (2.9)	0.8 [0.71, 0.99]	33 (1.6)	0.5 [0.32, 0.63]
ICH	1 (0.7)	0.3 [0.04, 2.00]	17 (0.9)	0.3 [0.17, 0.43]	31 (0.6)	0.2 [0.13, 0.26]	12 (0.6)	0.2 [0.09, 0.29]
Any GI bleeding	7 (5.2)	2.1 [0.98, 4.31]	97 (4.9)	1.6 [1.28, 1.91]	183 (3.8)	1.1 [0.95, 1.27]	44 (2.1)	0.6 [0.45, 0.81]
Major GI bleeding	5 (3.7)	1.4 [0.60, 3.48]	42 (2.1)	0.7 [0.49, 0.90]	59 (1.2)	0.3 [0.27, 0.45]	13 (0.6)	0.2 [0.10, 0.31]

*Note:* Definition of CKD stages (eGFR, CKD‐EPI Formula): Stage 4: eGFR ≥ 15 to < 30 mL/min/1.73 m^2^, Stage 3: eGFR ≥ 30 to < 60 mL/min/1.73 m^2^, Stage 2: eGFR ≥ 60 to < 90 mL/min/1.73 m^2^, Stage 1: eGFR ≥ 90 mL/min/1.73 m^2^. Definition of CV‐death sensitivity: Death due to CV cause or bleeding, or if a stroke, TIA, SEE, PE, MI, VTE or major bleeding occurred within 30 days before death and the reason for death is unknown or missing.

Abbreviations: CI, confidence interval; CKD, chronic kidney disease; CrCl, derived creatinine clearance (Cockgroft‐Gault); CV, cardiovascular; GI, gastrointestinal; ICH, intracranial haemorrhage; SEE, systemic embolism event; TIA, transient ischemic attack.

^a^ Category of missing CKD is not shown.

**FIGURE 2:**
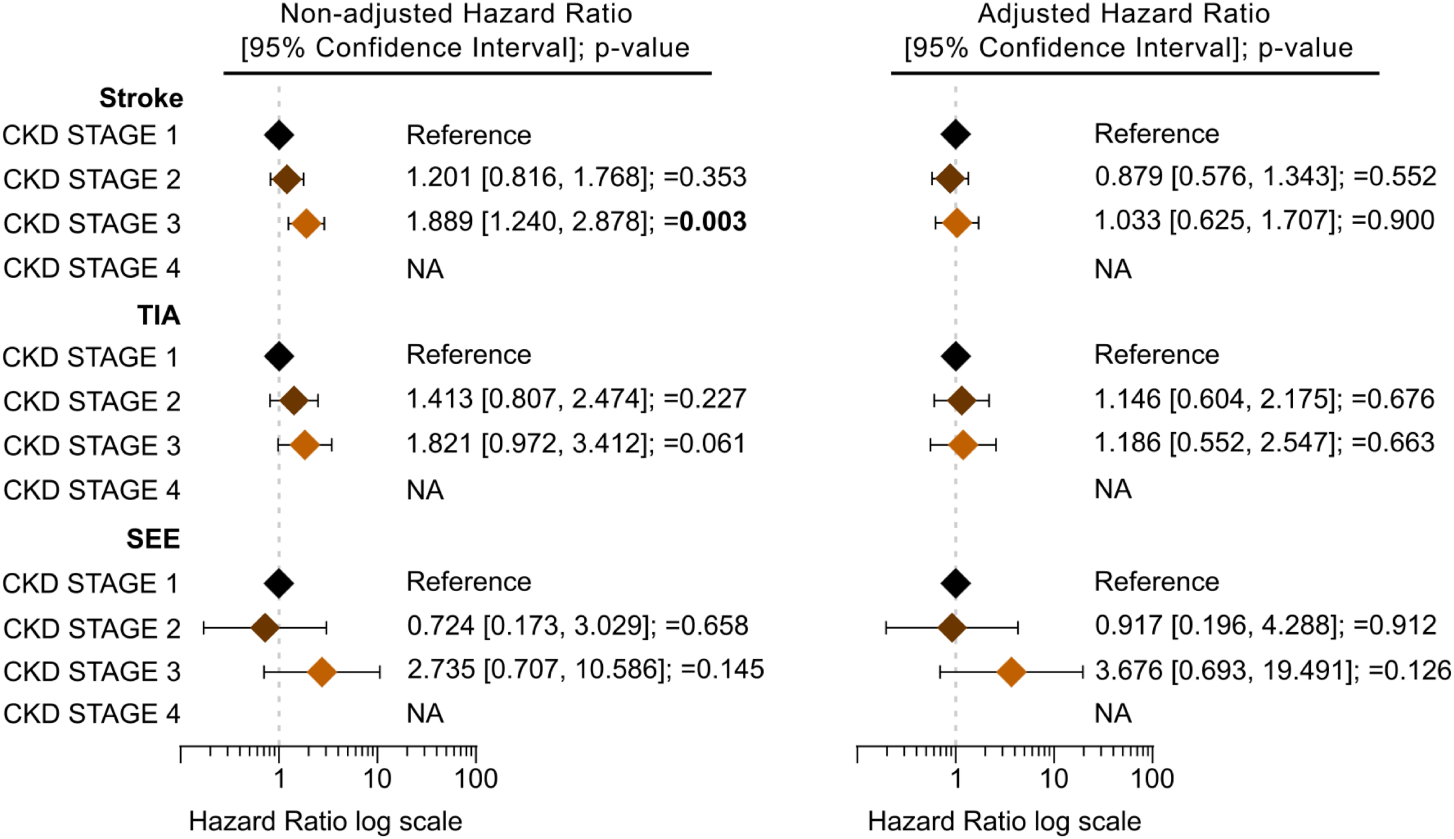
Non‐adjusted and adjusted HR comparing renal stages versus the reference stage (≥ 90 mL/min) for effectiveness outcomes of interest in patients from ETNA‐AF Europe 4‐year follow‐up on label‐recommended edoxaban dose (30 or 60 mg) (FAS, *N* = 9114). Hazard ratios were adjusted for age, sex, body weight and CHA_2_DS_2_‐VASc score and modified HAS‐BLED. Definition of CKD stages (eGFR, CKD‐EPI Formula): Stage 4: eGFR ≥ 15 to < 30 mL/min/1.73 m^2^, Stage 3: eGFR ≥ 30 to < 60 mL/min/1.73 m^2^, Stage 2: eGFR ≥ 60 to < 90 mL/min/1.73 m^2^, Stage 1: eGFR > 90 mL/min/1.73 m^2^. For categories with < 5 observations no HR, CIs and *p*‐values are provided. AF, atrial fibrillation; CI, confidence interval; CKD, chronic kidney disease; eGFR, estimated glomerular filtration rate; FAS, full analysis set; HR, hazard ratio; NA, not assessable; SEE, systemic embolism event.

### 3.3 Safety and Bleeding Outcomes per CKD Stage

The annualized rates of CV death (sensitivity), all‐cause death and bleeding events in patients on recommended edoxaban doses varied across CKD stages, with higher rates observed in patients with more advanced renal impairment (Table 2). Annualized rates of CV death (sensitivity) were markedly high in CKD Stage 4 patients (5.9% per year [95% CI: 3.86–9.08]) versus Stage 1 (0.4% per year [95% CI: 0.28–0.58]). All‐cause mortality showed the most pronounced difference, with CKD Stage 4 patients experiencing markedly higher rates (16.9% per year [95% CI: 13.14–21.79]) versus Stage 1 (2.0% per year [95% CI: 1.71–2.36]). The annual rate of any bleeding was highest in CKD Stage 4 (4.6% per year [95% CI: 2.75–7.58]), decreasing progressively across stages to 2.2% in Stage 1 (95% CI: 1.89–2.59; Table 2 and Table S2).

Major bleeding followed a similar trend, with annualized rates of 2.0% (95% CI: 0.97–4.25) in CKD Stage 4, to 0.5% (95% CI: 0.32–0.63) in Stage 1. ICH was rare (CKD Stage 4 [0.3%, 95% CI: 0.04–2.00] and Stage 1 [0.2%, 95% CI: 0.09–0.29]). GI bleeding, both any and major, also showed a declining trend, with any GI bleeding occurring at a rate of 2.1% (95% CI: 0.98–4.31) in CKD Stage 4 and 0.6% (95% CI: 0.45–0.81) in CKD Stage 1, while major GI bleeding was 1.4% (95% CI: 0.60–3.48) in Stage 4 and 0.2% (95% CI: 0.10–0.31) in Stage 1.

Non‐adjusted CV death (sensitivity) risk increased with worsening CKD stages (HR [95% CI]: Stage 4: 14.450 [8.270–25.247], Stage 3: 4.672 [3.312–6.969], Stage 2: 2.066 [1.394–3.062], all *p* < 0.001 vs. Stage 1). All‐cause death also showed a similar trend. Additionally, worsening renal function was associated with a statistically significant increased risk for adverse events at a nominal level: any bleeding, major bleeding, any GI bleeding and major GI bleeding versus patients with Stage 1 CKD (non‐adjusted HR: Figure 3, left panel). When outcomes were adjusted for age, sex, body weight and clinical risk scores, the risk patterns remained similar for CV death (sensitivity) (HR [95% CI]: Stage 4: 6.967 [3.748–12.951], *p* < 0.001; Stage 3: 2.114 [1.316–3.394], *p* = 0.002), though for Stage 2 no statistically robust difference was shown. A similar pattern was observed for all‐cause death (adjusted HR: Figure 3, right panel).

**FIGURE 3:**
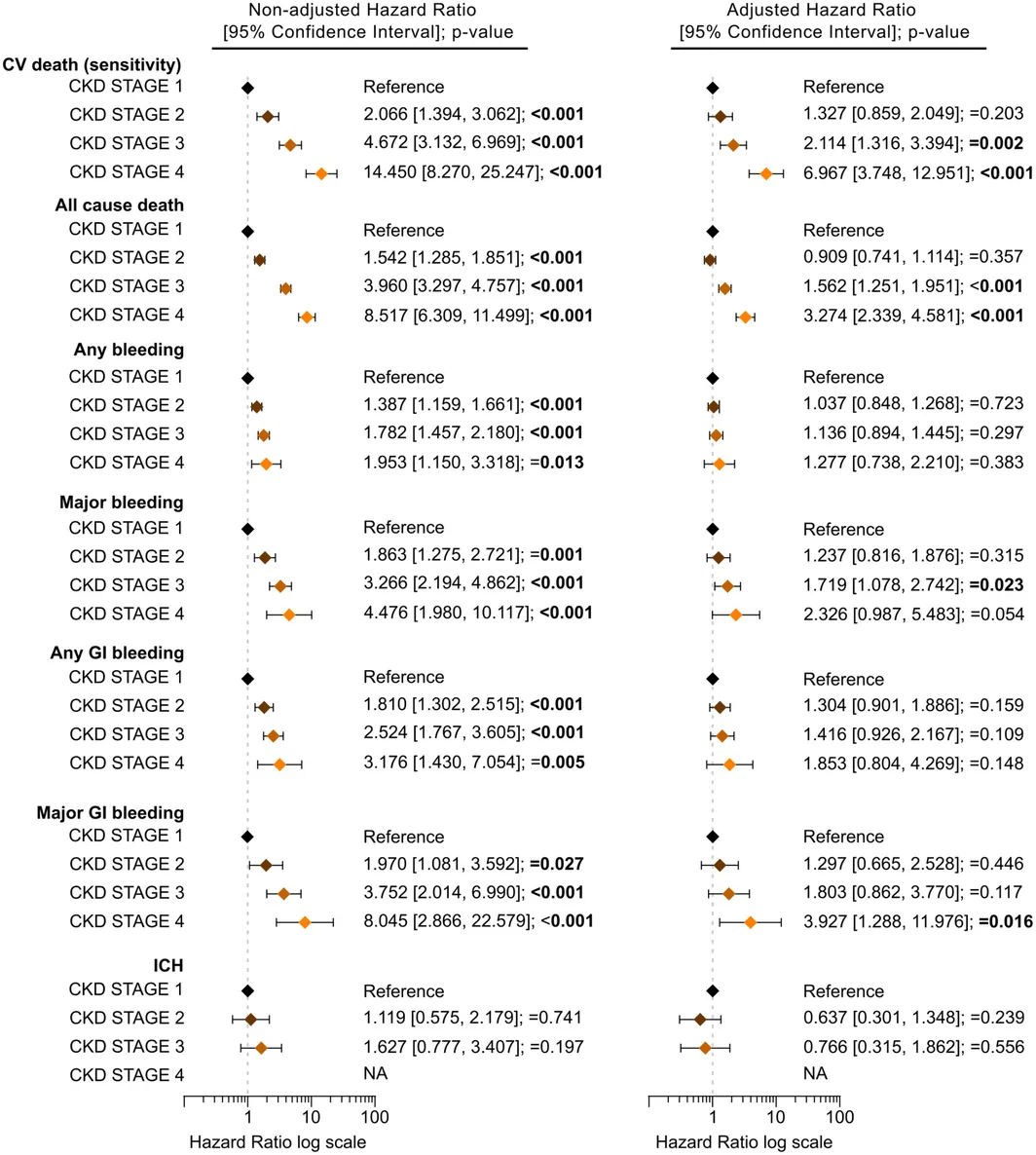
Non‐adjusted and adjusted HR comparing CKD Stages versus the reference Stage 1 (≥ 90 mL/min) for safety outcomes of interest in patients from ETNA‐AF Europe 4‐year follow‐up on label‐recommended edoxaban dose (30 or 60 mg; FAS, *N* = 9114). Hazard ratios were adjusted for age, sex, body weight and CHA_2_DS_2_‐VASc score and modified HAS‐BLED. Definition of CKD stages (eGFR, CKD‐EPI Formula): Stage 4: eGFR ≥ 15 to < 30 mL/min/1.73 m^2^, Stage 3: EGFR ≥ 30 to < 60 mL/min/1.73 m^2^, Stage 2: eGFR ≥ 60 to < 90 mL/min/1.73 m^2^, Stage 1: eGFR ≥ 90 mL/min/1.73 m^2^. Definition of CV‐death sensitivity: Death due to CV cause or bleeding, or if a stroke, TIA, SEE, PE, MI, VTE or major bleeding occurred within 30 days before death and the reason for death is unknown or missing. For categories with < 5 observations no HR and CIs are provided. CI, confidence interval; CKD, chronic kidney disease; CrCl, derived creatinine clearance; CV, cardiovascular; FAS, full analysis set; GI, gastrointestinal; HR, hazard ratio; ICH, intracranial haemorrhage; MI, myocardial infarction; NA, not assessable; PE, pulmonary embolism; TIA, transient ischemic attack; VTE, venous thromboembolism.

The adjusted risk for most bleeding events were not show a statistically robust difference versus the risk of patients with Stage 1 with notable exceptions of major bleeding in Stage 3 (HR [95% CI]: 1.719 [1.078–2.742], *p* = 0.023) and major GI bleeding in Stage 4 (HR [95% CI]: 3.927 [1.288–11.976], *p* = 0.016). For any bleeding, risks for patients with Stages 2, 3 and 4 were not significantly different at a nominal level versus that in Stage 1 (HR [95% CI]: Stage 2, 1.037 [0.848, 1.268], *p* = 0.723; Stage 3, 1.136 [0.894,1.445], *p* = 0.297; Stage 4, 1.277 [0.738, 2.210], *p* = 0.383). Similar results were seen for ICH and any GI bleeding (Figure 3, right panel).

## 4 Discussion

Because edoxaban is primarily excreted through the renal route (> 50% renal clearance), it is imperative to know the effect of renal function on the safety and effectiveness of the drug in different degrees of renal impairment. This ETNA‐AF‐Europe 4‐year follow‐up sub‐analysis provides critical insights in patients with AF, specifically highlighting the influence of CKD stages on clinical outcomes.

In ETNA‐AF‐Europe 4‐year follow‐up, annualized stroke rates were highest in patients with CKD Stage 3 (0.9% per year). Rates of TIA and SEE followed a similar pattern, peaking in patients with CKD Stage 4 (0.9% and 0.6%, respectively). This pattern aligns with the higher CHA_2_DS_2_‐VASc scores observed in advanced CKD stages, reflecting the cumulative CV risk burden in this population [6, 16, 17, 18]. When compared to patients with CKD Stage 1, worsening renal function was associated with a significantly higher risk (at a nominal level) of stroke in patients with CKD Stage 3, along with a higher TIA and SEE risk in patients with CKD Stage 4. When adjusted for confounding factors, there were no statistically robust differences in risk of these outcomes for Stages 2, 3 and 4 as versus Stage 1. Altogether, these observations suggest that, while renal impairment increases baseline risk for stroke and related outcomes, label‐recommended edoxaban dosing may provide adequate thromboembolic protection across all CKD stages.

Results from the ETNA‐AF Europe 4‐year follow‐up showed that advanced CKD stages were associated with markedly increased annual rates for bleeding, including any bleeding, major bleeding and GI bleeding. The annual rate of major bleeding in patients with CKD Stage 4 (2.0% per year) was four times that in patients with CKD Stage 1 (0.5% per year).

The rate of all‐cause death was progressively higher in patients with advanced CKD, with CKD Stage 4 patients experiencing an annual all‐cause mortality of 16.9% per year, versus 2.0% per year in CKD Stage 1. A similar pattern was also observed for CV death (sensitivity). The high death rates in advanced CKD stages are likely multifactorial, reflecting not only the risks associated with AF and anticoagulation therapy but also the broader impact of age, frailty and comorbidities such as heart failure and hypertension [19].

Advanced stages of CKD are associated with increased CV risk and the risk of AF is 10–20 times higher in patients with end‐stage renal disease than in the normal population [20]. Even after adjusting for confounding variables, the ETNA‐AF‐Europe 4‐year follow‐up data showed that patients with CKD Stage 4 had a nearly 3.5‐fold higher risk of all‐cause death and a 7‐fold higher risk of CV death (sensitivity) compared with patients with Stage 1, suggesting that renal impairment is a strong independent predictor of mortality. ETNA‐AF‐Europe showed that, if the label‐recommended dose of 30 mg was followed for patients with CKD Stage 4, although the risks for all‐cause and CV death (sensitivity) remained high, the risk for bleeding events was not different versus CKD Stage 1 patients. These findings support the use of the 60 mg dose of edoxaban in patients with mild‐to‐moderate renal impairment and suggest that, when patients with CKD receive appropriate labelled doses of edoxaban, renal function does not substantially alter the safety and effectiveness profile of edoxaban for atrial fibrillation. However, the persistently higher mortality observed with worsening CKD likely reflects the underlying disease burden rather than differences in treatment‐related outcomes.

Although not a direct comparison, the observational outcomes from ETNA‐AF‐Europe can be contextualized with the ENGAGE‐TIMI randomized study, showing a pattern of increased age, lower body weight, frequent dose reductions, higher perceived frailty and higher prevalence of CV and bleeding‐related risk factors in patients with greater kidney impairment [11, 14, 21]. Both studies show that reduced renal function had higher annualized rates of stroke or SEE, CV death and major bleeding. This underpins the importance of considering renal function when evaluating the effectiveness and safety of edoxaban in patients with AF. In the ETNA‐AF‐Europe overall study population, most patients with CKD Stage 4 had their dose reduced to 30 mg from 60 mg as per label recommendations, showing adherence to dosing recommendations [7, 22]. In ENGAGE‐TIMI, when compared to warfarin, the efficacy of a higher‐dose edoxaban regimen was similar for the prevention of stroke or SEE, with no modification of the treatment effect by renal function [14]. Similar to the observation here, in ENGAGE‐TIMI major bleeding and risk for CV death was higher in patients with moderate renal dysfunction at baseline (CrCl 30–50 mL/min; 4.0% per year) versus patients with mild or no renal dysfunction (CrCl > 50 mL/min; 2.5% per year) [14]. To note, patients with severe renal impairment were excluded from ENGAGE‐TIMI: thus, ETNA‐AF‐Europe bridged the knowledge gap for this patient population. Previous data have expanded on this concept, demonstrating that CKD frequently clusters not only with older age, lower body weight and higher perceived frailty, but also with a prior history of stroke and bleeding, identifying a subgroup of patients who are particularly complex to manage given their concurrently elevated ischemic and haemorrhagic risk and narrow therapeutic window for oral anticoagulation [23]. This area remains an active area of research, with novel investigational anticoagulants and non‐pharmacological approaches, such as left atrial appendage occlusion, currently being explored as potential alternatives for these particularly high‐risk patients.

ETNA‐AF‐Europe provides data from a large, diverse cohort of AF patients with extended follow‐up, offering a unique opportunity to evaluate long‐term safety and effectiveness outcomes of edoxaban in real‐world settings. However, observational design introduces potential biases, including potentially unmeasured confounding variables and the difficulty in establishing causality. Furthermore, uncontrolled factors such as patient adherence, physician prescribing patterns and concomitant medications might have influenced outcomes [11]. Since age is a direct component of the CKD‐EPI formula used to calculate eGFR and was also included as a covariate in the adjusted models, part of the effect of renal function on outcomes may have been attenuated by adjustment for age; disentangling the independent contribution of age from that of renal function itself remains challenging in this type of analysis and should be considered when interpreting the adjusted results. For certain outcomes, such as stroke, ICH, SEE and TIA, the small number of patients with advanced CKD stages and insufficient number of observations limited the interpretation of data for these events. Nevertheless, our conclusions are based on a reasonably high sample size for up to CKD Category 3. Among the 162 Stage 4 CKD patients enrolled, 135 (83.3%) received SmPC‐recommended edoxaban doses and were included in this analysis, a proportion comparable to Stage 1 and higher than Stages 2 and 3, arguing against substantial selection of a lower‐risk subset. Nonetheless, given the small absolute number of Stage 4 patients, outcomes in this subgroup should be interpreted with caution. In general, it has to be considered that this was a post hoc analysis that was not adjusted for any multiple testing. All *p*‐values are nominal, provided for descriptive purposes only and should be interpreted with caution. Despite its limitations, the observational real‐world evidence ETNA‐AF‐Europe 4‐year follow‐up helps contextualize clinical trial findings into routine practice while considering the distinct characteristics of real‐world populations.

## 5 Conclusions

The ETNA‐AF‐Europe 4‐year follow‐up data presented here highlights the impact of renal function on the safety and effectiveness of edoxaban in patients with AF. After adjusting for various influencing factors, the safety risks with advanced CKD stages did not differ from the risks with Stage 1 CKD, thus confirming the effectiveness and safety profile of edoxaban in patients with AF when following label‐recommended dosages (30 or 60 mg), irrespective of renal disease stages. By analysing the alignment between real‐world and randomized controlled trial data, this ETNA‐AF‐Europe 4‐year follow‐up sub‐analysis refines our understanding of anticoagulation management in AF patients with varying degrees of renal impairment, ultimately providing insights that can support treatment strategies in AF patients with renal impairment.

## Author Contributions

R.D.C. contributed to conceptualization, methodology, supervision, writing, review and editing. E.‐M.F. contributed to conceptualization, methodology, data curation, formal analysis, writing, review and editing. J.N. contributed to conceptualization, project administration, writing, review and editing. M.L. contributed to conceptualization, methodology, project management, writing, review and editing. P.K. contributed to conceptualization, methodology, writing, review and editing. C.Z. contributed to conceptualization, methodology, writing, review and editing. All authors read and approved the final manuscript.

## Funding

This work was supported by Daiichi Sankyo Europe GmbH.

## Disclosure

All authors take responsibility for all aspects of reliability and freedom from bias of the data presented and their discussed interpretation.

## Conflicts of Interest

R.D.C. declares Fees, honoraria and research funding from Daiichi Sankyo, Sanofi‐Aventis, Boehringer Ingelheim, Bayer, BMS/Pfizer, Novartis, Merck, Portola, Roche, AstraZeneca, Menarini, Guidotti, Milestone, Amarin, Noventure and Amgen. E.‐M.F., J.N. and M.L. are employees of Daiichi Sankyo Europe GmbH. P.K. receives research support for basic, translational and clinical research projects from European Union Big‐Data@Heart (Grant Agreement EU IMI 116 074), CATCH ME (Grant Agreement ID: 633 196), AFFECT‐EU (Grant Agreement ID: 847 770), Leducq Foundation, Medical Research Council (UK), German Centre for Cardiovascular Research supported by the German Ministry of Education and Research, and from several drug and device companies. He has received honoraria from several such companies in the past, but not in the last 3 years. He is listed as an inventor on two patents held by the University of Birmingham (Atrial Fibrillation Therapy WO 2 015 140 571, Markers for Atrial Fibrillation WO 2 016 012 783). P.K. is employed as Director of the Department of Cardiology, University Heart and Vascular Centre UKE Hamburg, and a Professor of Cardiovascular Medicine (part time), University of Birmingham, UK. He is a speaker on the board of AFNET, Germany, and a board member of the European Society of Cardiology. C.Z. declares no potential conflicts of interest.

## Supporting information


**Section S1:** CHA_2_DS_2_‐VASc score (heart failure [1 point], hypertension [1 point], ≥ 75 years [2 points], diabetes mellitus [1 point], stroke/TIA/SEE [2 points], vascular disease [1 point], age 65–74 years [1 point], female sex [1 point]). Modified HAS‐BLED (hypertension [1 point], creatinine clearance (CrCl) ≤ 80 mL/min or liver disease [1 or 2 points], stroke history [1 point], prior major bleeding or predisposition to bleeding [1 point], age of > 65 years [1 point], medication usage predisposing to bleeding or alcohol usage [1 or 2 points]).
**Section S2:** ETNA‐AF‐Europe enrolled 13,164 patients, with 11,780 patients having eGFR data, showing renal function across CKD stages: Stage 1 (*n* = 2491), Stage 2 (*n* = 6216), Stage 3 (*n* = 2908), Stage 4 (*n* = 162) and Stage 5 (*n* = 3) (Figure 1). For completeness and transparency, ETNA‐AF‐Europe overall study population showed consistent trends in age, body weight and frailty increasing with CKD severity (Table S1). CKD Stage 4 patients were older (mean age ~80 years) and frailer (32.7%). Dose‐reduction criteria (e.g., CrCl < 50 mL/min) and CHA_2_DS_2_‐VASc scores followed similar distributions, with higher scores (≥ 2) in severe CKD stages. Bleeding history and CV comorbidities also increased with CKD severity, while history of GI bleeding and stroke were consistently more prevalent in advanced stage.
**Table S1:** Baseline characteristics by renal stages for all patients (on‐ and off‐label) from ETNA‐AF Europe (full analysis set, *N* = 13,164).
**Table S2:** Absolute and relative frequencies, annual rates and confidence intervals of outcomes of interest by renal stages in all patients from ETNA‐AF Europe 4‐year follow‐up that were on label‐recommended edoxaban dose (30 mg [*N* = 2032] or 60 mg [7082]).

## Data Availability

The datasets presented in this article are not readily available due to technical/time limitations. Requests to access the datasets should be directed to https://vivli.org/ourmember/daiichi‐sankyo/.
