# Supplementary material for: Renal Function and 4‐Year Outcomes in Edoxaban‐Treated Patients: A Sub‐Analysis From the ETNA‐AF Europe Study
Source: Eur J Clin Invest. 2026 Oct 28;56(11):e70268. doi: 10.1111/eci.70268 (PMC13714686; doi:10.1111/eci.70268)
Supplement: Supplementary file 1 [file ECI-56-e70268-s001.docx]

**Section S1**

- CHA_2_DS_2_-VASc score (heart failure [1 point], hypertension [1 point], ≥75 years [2 points], diabetes mellitus [1 point], stroke/TIA/SEE [2 points], vascular disease [1 point], age 65 to 74 years [1 point], female sex [1 point])
- Modified HAS-BLED (hypertension [1 point], creatinine clearance (CrCl) ≤80 mL/min or liver disease [1 or 2 points], stroke history [1 point], prior major bleeding or predisposition to bleeding [1 point], age of >65 years [1 point], medication usage predisposing to bleeding or alcohol usage [1 or 2 points].

**Section S2**

ETNA-AF-Europe enrolled 13,164 patients, with 11,780 patients having eGFR data, showing renal function across CKD stages: Stage 1 (n=2,491), Stage 2 (n=6,216), Stage 3 (n=2,908), Stage 4 (n=162), and Stage 5 (n=3) **(Figure 1)**. For completeness and transparency, ETNA-AF-Europe overall study population showed consistent trends in age, body weight, and frailty increasing with CKD severity **(Table S1)**. CKD Stage 4 patients were older (mean age ~80 years), and frailer (32.7%). Dose-reduction criteria (e.g., CrCl <50 mL/min) and CHA_2_DS_2_-VASc scores followed similar distributions, with higher scores (≥2) in severe CKD stages. Bleeding history and CV comorbidities also increased with CKD severity, while history of GI bleeding and stroke were consistently more prevalent in advanced stages.

|  | **Total** | **STAGE 5** (N=3) | **STAGE 4** (N=162) | **STAGE 3** (N=2,908) | **STAGE 2** (N=6,216) | **STAGE 1** (N=2,491) |
| --- | --- | --- | --- | --- | --- | --- |
| **N** (%) | 13,164 (100.0) | 3 (<0.1) | 162 (1.4) | 2,908 (24.7) | 6,216 (52.8) | 2,491 (21.1) |
| **Male**, n (%) | 7,461 (56.7) | 3 (100.0) | 57 (35.2) | 1,367 (47.0) | 3,586 (57.7) | 1,617 (64.9) |
| **Age (years)**, mean (SD) | 73.6 (9.5) | 66.3 (11.2) | 79.5 (7.8) | 78.7 (7.6) | 74.2 (8.6) | 66.6 (9.3) |
| ≥ 75 years old | 6,706 (50.9) | 1 (33.3) | 124 (76.5) | 2,152 (74.0) | 3,304 (53.2) | 477 (19.1) |
| **Body weight (kg)**, mean (SD) | 80.9 (17.2) | 83.3 (11.5) | 78.1 (18.0) | 78.5 (16.4) | 80.4 (16.6) | 84.0 (18.9) |
| **BMI (kg/m^2^)**, mean (SD) | 28.1 (5.1) | 25.6 (3.6) | 28.3 (5.4) | 28.1 (5.1) | 27.9 (4.9) | 28.5 (5.6) |
| **Patients with dose reduction criteria**, n (%) | | | | | | |
| CrCl <50mL/min | 2,454 (20.9) | 3 (100.0) | 159 (98.1) | 1,793 (61.9) | 486 (7.9) | 1 (<0.1) |
| Body weight ≤ 60kg | 1,319 (10.4) | 0 | 26 (16.4) | 357 (12.5) | 631 (10.5) | 209 (8.6) |
| Concomitant use of P-gp inhibitors | 353 (2.8) | 0 | 5 (3.2) | 91 (3.2) | 150 (2.5) | 68 (2.8) |
| **Edoxaban 60mg**, n (%) | 9,617 (75.9) | 1 (33.3) | 17 (11.0) | 1,226 (43.6) | 5,054 (84.3) | 2,225 (92.6) |
| **Edoxaban recommended**, n (%) | 9,114 (72.0) | 0 | 135 (87.1) | 1,985 (70.7) | 4,855 (81.1) | 2,064 (86.0) |
| 30mg | 2,032 (16.1) | 0 | 135 (87.1) | 1,277 (45.5) | 468 (7.8) | 89 (3.7) |
| 60mg | 7,082 (56.0) | 0 | 0 | 708 (25.2) | 4,387 (73.3) | 1,975 (82.3) |
| **CHA_2_DS_2_-VASc (cal.)**, mean (SD) | 3.2 (1.4) | 1.3 (1.5) | 4.4 (1.2) | 3.9 (1.3) | 3.2 (1.4) | 2.5 (1.4) |
| CHA_2_DS_2_-VASc = 0, n (%) | 251 (2.0) | 1 (33.3) | 0 | 5 (0.2) | 80 (1.3) | 130 (5.3) |
| CHA_2_DS_2_-VASc = 1, n (%) | 1,222 (9.5) | 1 (33.3) | 0 | 70 (2.5) | 542 (8.9) | 467 (19.2) |
| CHA_2_DS_2_-VASc ≥ 2, n (%) | 11,369 (88.5) | 1 (33.3) | 157 (100.0) | 2,746 (97.3) | 5,463 (89.8) | 1,837 (75.5) |
| **HAS-BLED (cal.)**, mean (SD) | 2.5 (1.0) | 2.0 (1.0) | 3.2 (0.7) | 3.0 (0.7) | 2.6 (0.9) | 1.7 (0.9) |
| **Frailty-categorized by physician**, n (%) | 1,410 (10.7) | 1 (33.3) | 53 (32.7) | 584 (20.1) | 620 (10.0) | 114 (4.6) |
| **History of CV disease**, n (%) | | | | | | |
| Hypertension | 10,155 (77.1) | 2 (66.7) | 144 (88.9) | 2,419 (83.2) | 4,694 (75.5) | 1,799 (72.2) |
| Congestive heart failure | 2,042 (15.5) | 0 | 55 (34.0) | 691 (23.8) | 895 (14.4) | 289 (11.6) |
| Myocardial infarction | 567 (4.3) | 0 | 10 (6.2) | 176 (6.1) | 261 (4.2) | 100 (4.0) |
| Valvular disease | 1,870 (14.2) | 0 | 44 (27.2) | 556 (19.1) | 859 (13.8) | 253 (10.2) |
| Peripheral artery disease | 432 (3.3) | 0 | 11 (6.8) | 148 (5.1) | 184 (3.0) | 63 (2.5) |
| **History of diabetes mellitus**, n (%) | 2,881 (21.9) | 0 | 73 (45.1) | 842 (29.0) | 1,207 (19.4) | 498 (20.0) |
| **History of stroke and ICH**, n (%) | | | | | | |
| Ischemic stroke | 697 (5.3) | 0 | 14 (8.6) | 164 (5.6) | 366 (5.9) | 116 (4.7) |
| Stroke, unknown | 73 (0.6) | 0 | 1 (0.6) | 20 (0.7) | 31 (0.5) | 12 (0.5) |
| Transient Ischemic Attack | 449 (3.4) | 0 | 3 (1.9) | 114 (3.9) | 233 (3.7) | 72 (2.9) |
| Intracranial Hemorrhage | 62 (0.5) | 0 | 1 (0.6) | 21 (0.7) | 27 (0.4) | 9 (0.4) |
| **History of bleeding**, n (%) | | | | | | |
| Any bleeding | 416 (3.2) | 0 | 8 (4.9) | 132 (4.5) | 195 (3.1) | 50 (2.0) |
| GI bleeding (Major or CRNM) | 108 (0.8) | 0 | 4 (2.5) | 43 (1.5) | 48 (0.8) | 6 (0.2) |
| Major | 118 (0.9) | 0 | 2 (1.2) | 41 (1.4) | 51 (0.8) | 16 (0.6) |
| CRNM | 140 (1.1) | 0 | 3 (1.9) | 46 (1.6) | 69 (1.1) | 12 (0.5) |
| Minor | 159 (1.2) | 0 | 3 (1.9) | 46 (1.6) | 74 (1.2) | 24 (1.0) |
| **Current AF type**, n (%) | | | | | | |
| Paroxysmal | 7,083 (53.9) | 2 (66.7) | 76 (46.9) | 1,386 (47.8) | 3,348 (54.0) | 1,435 (57.7) |
| Persistent | 3,175 (24.2) | 1 (33.3) | 37 (22.8) | 704 (24.3) | 1,544 (24.9) | 620 (24.9) |
| Long/standing persistent | 322 (2.5) | 0 | 7 (4.3) | 86 (3.0) | 143 (2.3) | 61 (2.5) |
| Permanent | 2,559 (19.5) | 0 | 42 (25.9) | 726 (25.0) | 1,169 (18.8) | 371 (14.9) |

**Table S1**: Baseline characteristics by renal stages for all patients (on- and off-label) from ETNA-AF Europe (Full Analysis Set, N=13,164)*. *Category of missing CKD not shown. Definition of CKD stages (eGFR, CKD-EPI Formula): STAGE 5: eGFR <15 mL/min/1.73 m^2^, STAGE 4: eGFR ≥15 to <30 mL/min/1.73 m^2^, STAGE 3: eGFR ≥30 to <60 mL/min/1.73 m^2^, STAGE 2: eGFR ≥60 to <90 mL/min/1.73 m^2^, STAGE 1: eGFR ≥ 90 mL/min/1.73 m^2^. Abbreviations: AF, atrial fibrillation; BMI, body mass index; CHA2DS2-VASc, congestive heart failure (1 point), hypertension (1 point), age ≥75 (2 points), diabetes (1 point), stroke/TIA/SEE (2 points), vascular disease (1 point), age 65 to 74 (1 point), and female sex (1 point); cal., calculated; CKD-EPI, Chronic kidney disease-epidemiology collaboration; CrCl, derived creatinine clearance (Cockgroft-Gault); CRNM, clinically relevant non-major; CV, cardiovascular; GFR, Glomerular filtration rate; GI, gastrointestinal; HAS-BLED, hypertension (1 point), CrCl≤80mL/min or liver disease (1 or 2 points), stroke history (1 point), bleeding history or predisposition (1 point), age of >65 years (1 point), medication usage predisposing to bleeding or alcohol usage (1 or 2 points); ICH, intracranial hemorrhage; P-gp, P-glycoprotein 1 inhibitors requiring edoxaban dose reduction (cyclosporine, dronedarone, erythromycin, ketoconazole); SD, standard deviation; SEE, systemic embolism event; TIA, transient ischemic attack.

|  | **CKD STAGE 4** (N=135) | | **CKD STAGE 3** (N=1,985) | | **CKD STAGE 2** (N=4,855) | | **CKD STAGE 1** (N=2,064) | |
| --- | --- | --- | --- | --- | --- | --- | --- | --- |
|  | n (%) | Annual rates  % [95% CI] | n (%) | Annual rates  % [95% CI] | n (%) | Annual rates  % [95% CI] | n (%) | Annual rates  % [95% CI] |
| **Stroke** |  |  |  |  |  |  |  |  |
| 30 mg | 2 (1.5) | 0.6 [0.14, 2.28] | 41 (3.2) | 1.1 [0.78, 1.43] | 13 (2.8) | 0.9 [0.51, 1.52] | 3 (3.4) | 1.1 [0.34, 3.29] |
| 60 mg | - | - | 16 (2.3) | 0.7[0.41, 1.09] | 84 (1.9) | 0.5 [0.44, 0.68] | 32 (1.6) | 0.5 [0.32, 0.64] |
| **TIA** |  |  |  |  |  |  |  |  |
| 30 mg | 3 (2.2) | 0.9 [0.28, 2.66] | 16 (1.3) | 0.4 [0.25, 0.67] | 5 (1.1) | 0.3 [0.14, 0.81] | - | - |
| 60 mg | - | - | 9 (1.3) | 0.4 [0.19, 0.72] | 47 (1.1) | 0.3 [0.23, 0.41] | 16 (0.8) | 0.2 [0.14, 0.37] |
| **SEE** |  |  |  |  |  |  |  |  |
| 30 mg | 2 (1.5) | 0.6 [0.14, 2.27] | 4 (0.3) | 0.1 [0.04, 0.27] | 2 (0.4) | 0.1 [0.03, 0.54] | - | - |
| 60 mg | - | - | 3 (0.4) | 0.1 [0.04, 0.38] | 3 (<0.1) | 0.0 [0.01, 0.06] | 3 (0.2) | 0.0 [0.01, 0.13] |
| **CV death (sensitivity)** | |  |  |  |  |  |  |  |
| 30 mg | 21 (15.6) | 5.9 [3.86, 9.08] | 98 (7.7) | 2.5 [2.05, 3.04] | 27 (5.8) | 1.8 [1.24, 2.64] | 1 (1.1) | 0.4 [0.05, 2.50] |
| 60 mg | - | - | 23 (3.2) | 0.9 [0.63, 1.43] | 116 (2.6) | 0.7 [0.62, 0.90] | 29 (1.5) | 0.4 [0.28, 0.59] |
| **All-cause death** |  |  |  |  |  |  |  |  |
| 30 mg | 60 (44.4) | 16.9 [13.14, 21.79] | 410 (32.1) | 10.4 [9.48, 11.50] | 106 (22.6) | 7.1 [5.87, 8.60] | 20 (22.5) | 7.1 [4.55, 10.93] |
| 60 mg | - | - | 93 (13.1) | 3.8 [3.13, 4.70] | 420 (9.6) | 2.7 [2.46, 2.98] | 128 (6.5) | 1.8 [1.52, 2.15] |
| **Any bleeding** |  |  |  |  |  |  |  |  |
| 30 mg | 15 (11.1) | 4.6 [2.75, 7.58] | 149 (11.7) | 4.0 [3.43, 4.73] | 48 (10.3) | 3.4 [2.57, 4.53] | 10 (11.2) | 3.7 [1.98, 6.84] |
| 60 mg | - | - | 91 (12.9) | 4.1 [3.31, 4.99] | 446 (10.2) | 3.1 [2.78, 3.35] | 146 (7.4) | 2.2 [1.83, 2.54] |
| **Major bleeding** |  |  |  |  |  |  |  |  |
| 30 mg | 7 (5.2) | 2.0 [0.97, 4.25] | 61 (4.8) | 1.6 [1.23, 2.03] | 16 (3.4) | 1.1 [0.67, 1.78] | 3 (3.4) | 1.1 [0.34, 3.29] |
| 60 mg | - | - | 31 (4.4) | 1.3 [0.92, 1.86] | 125 (2.8) | 0.8 [0.68, 0.97] | 30 (1.5) | 0.4 [0.30, 0.61] |
| **ICH** |  |  |  |  |  |  |  |  |
| 30 mg | 1 (0.7) | 0.3 [0.04, 2.00] | 11 (0.9) | 0.3 [0.16, 0.51] | 2 (0.4) | 0.1 [0.03, 0.54] | 1 (1.1) | 0.4 [0.05, 2.50] |
| 60 mg | - | - | 6 (0.8) | 0.2 [0.11, 0.55] | 29 (0.7) | 0.2 [0.13, 0.27] | 11 (0.6) | 0.2 [0.09, 0.28] |
| **Any GI bleeding** |  |  |  |  |  |  |  |  |
| 30 mg | 7 (5.2) | 2.1 [0.98, 4.31] | 66 (5.2) | 1.7 [1.36, 2.20] | 22 (4.7) | 1.5 [1.00, 2.30] | 2 (2.2) | 0.7 [0.18, 2.84] |
| 60 mg | - | - | 31 (4.4) | 1.3 [0.92, 1.86] | 161 (3.7) | 1.1 [0.91, 1.24] | 42 (2.1) | 0.6 [0.44, 0.81] |
| **Major GI bleeding** |  |  |  |  |  |  |  |  |
| 30 mg | 5 (3.7) | 1.4 [0.60, 3.48] | 31 (2.4) | 0.8 [0.56, 1.13] | 6 (1.3) | 0.4 [0.18, 0.90] | 1 (1.1) | 0.4 [0.05, 2.51] |
| 60 mg | - | - | 11 (1.6) | 0.5 [0.25, 0.83] | 53 (1.2) | 0.3 [0.26, 0.45] | 12 (0.6) | 0.2 [0.10, 0.30] |

**Table S2:** Absolute and relative frequencies, annual rates and confidence intervals of outcomes of interest by renal stages in all patients from ETNA-AF Europe 4‑year follow-up that were on label-recommended edoxaban dose (30 mg [N=2,032] or 60 mg [7,082])*. *Category of missing CKD is not shown. Definition of CKD stages (eGFR, CKD-EPI Formula): STAGE 4: eGFR ≥15 to <30 mL/min/1.73 m^2^, STAGE 3: eGFR ≥30 to <60 mL/min/1.73 m^2^, STAGE 2: eGFR ≥60 to <90 mL/min/1.73 m^2^, STAGE 1: eGFR ≥ 90 mL/min/1.73 m^2^. Definition of CV-death sensitivity: Death due to CV cause or bleeding, or if a stroke, TIA, SEE, PE, MI, VTE, or major bleeding occurred within 30 days before death and the reason for death is unknown or missing. Abbreviations: CI, confidence interval; CKD, chronic kidney disease; CrCl, derived creatinine clearance (Cockgroft-Gault); CV, cardiovascular; GI, gastrointestinal; ICH, intracranial haemorrhage; SEE, systemic embolism event; TIA, transient ischemic attack.
